# Complex Dynamics of Photoinduced Mass Transport and Surface Relief Gratings Formation

**DOI:** 10.3390/nano9030352

**Published:** 2019-03-04

**Authors:** Grzegorz Pawlik, Tomasz Wysoczanski, Antoni C. Mitus

**Affiliations:** 1 Department of Theoretical Physics, Wroclaw University of Science and Technology, 50-370 Wroclaw, Poland; antoni.mitus@pwr.edu.pl; 2 Nabycinska 5-7/52, 53-677 Wroclaw, Poland; t.wysoczanski17@gmail.com

**Keywords:** all-optical modulation, Surface Relief Grating, anomalous dynamics

## Abstract

The microscopic and semi-macroscopic mechanisms responsible for photoinduced mass transport in functionalized azo-polymers are far from deeply understood. To get some insight into those mechanisms on “microscopic” scale, we studied the directed photoinduced motion of single functionalized polymer chains under various types of polarized light illumination using Monte Carlo bond fluctuation model and our kinetic Monte Carlo model for photoinduced mass transport. We found sub-diffusive, diffusive and super-diffusive regimes of the dynamics of single chains at constant illumination and mostly super-diffusive regime for directed motion in the presence of the gradient of light intensity. This regime is more enhanced for long than for short chains and it approaches the ballistic limit for very long chains. We propose a physical picture of light-driven inscription of Surface Relief Gratings (SRG) as corresponding to a dynamical coexistence of normal and anomalous diffusion in various parts of the system. A simple continuous time random walk model of SRG inscription based on this physical picture reproduced the light-driven mass transport found in experiments as well as the fine structure of SRG.

## 1. Introduction

Thin films of azobenzene functionalized polymers [1] may develop a periodic surface corrugation pattern called Surface Relief Grating (SRG) when exposed to interfering polarized laser beams in a Degenerated Two Wave Mixing (DTWM) experiment [2,3]. The microscopic movements of polymer chain, resulting from light-induced multiple trans↔cis photoisomerization cycles of azobenzene dyes attached to the chains, promote a macroscopic mass transport at the surface of polymer thin film at temperatures far below the polymers glass transition temperatures $T_{g}$.

The origin of the photoinduced mass transport in thin layers of polymer matrix functionalized with azo-dyes remains unclear. Several theories have been formulated (see recent reviews [4,5] and Ref. [1]). They include a mean-field model [6], pressure gradient model [7,8], competition between photoexpansion and photocontraction [9], viscoelastic flow model [10], stochastic inchworm-like motion [11], gradient force models [12,13], Navier–Stokes dynamics [14,15], random walk model [16], stochastic models [17,18,19], light-induced softening [4,12], and others.

Some of the approaches to the problem of SRG inscription are based on the concept of directional photofluidization or light-induced plasticization [4,12,20,21,22,23]. The origin of photofluidization scenario goes back to Kumar [12], who formulated the concept of light-induced plasticization caused by reorientation of chromophores (via trans↔cis cycles). On the other hand, the analysis of experimental results reported in Refs. [4,20,24,25] leads to the conclusion [26] that there is no convincing experimental evidence of photofluidization. Instead, it is pointed out that the phenomenological orientation approach based on the effective potential provides results in a good agreement with experimental data for photodeformation of azobenzene-containing polymers [27,28,29,30,31,32,33,34]. The main conclusion of those papers is that reorientation of chromophores (via trans↔cis cycles) and subsequent polymer chains movements can produce strong stresses in an azo-polymer system, sufficient to generate lasting deformations in glassy phase observed in experiments [35,36]. The effective orientation potential can be applied to study the reorientation kinetics, deformation and mass transport of a large variety of azo-materials, e.g., amorphous, liquid-crystalline and cross-linked polymers, azobenzene-functionalized dendrimers and brushes and many others (see Ref. [37] and literature cited therein). Recently, the orientation approach is justified by a theoretical analysis of kinetics of photoisomerization and time evolution of ordering in azobenzene-containing materials [37].

Those studies are oriented towards the physical origin of the deformations, which enable the motion of the polymer chains. On the other hand, the characterization of the resulting dynamics of the chains is, at best, at its very early stage. Some preliminary studies of this aspect of SRG formation are reported in Refs. [11,17,18,19] (see Ref. [38] for more details). We have proposed a generic stochastic Monte Carlo (MC) Bond Fluctuation Model (BFM) [39] of the photoinduced mass transport in azo-polymers [38], which mimics the effects of multiple photoisomerization cycles of functionalized dyes in a host polymer matrix, in the presence of spatially inhomogeneous light illumination. The model does not break, on the “microscopic” level, the left–right symmetry along the direction of light modulation. Nevertheless, it correctly reproduces experimental effects such as a directed mass transfer from bright to dark places and the fine structure of SRG—an effect observed but either not discussed [40] or disregarded [41]—as well as the theoretical relation between the velocity v(x) of center of mass (CM) of a polymer chain and gradient of the light illumination I(x): $v\left(x\right)\propto -\frac{dI(x)}{dx}$, postulated on the macroscopic level [7,8,12,13]. Nevertheless, the “microscopic” origin of the photoinduced directed motion remains unclear, in part because of lack of knowledge about motion of single functionalized polymer chains illuminated with polarized laser light.

MC dynamics in the bulk of such a system is the combined result of dynamics of single chains, modified by steric interaction due to neighbouring chains in a dense system. Thus, the first step towards a characterization of this motion is an attempt to decompose it into simpler but important motions of single chains. Three main topics arise in this context: (i) classification of the dynamics in dense system (bulk) at constant illumination; (ii) classification of the dynamics of functionalized single chains at constant illumination; and (iii) classification of the dynamics of functionalized single chain in the presence of its gradient. They constitute the objectives of this study.

We point out that there is also another aspect to this study. Namely, if a sound physical picture of the dynamics of single chains emerges, then it might constitute a reliable starting point for the construction of simple models of physical phenomena where photoinduced mass transport of azo-dye functionalized polymers plays the central role. This topic is discussed in the last part of the paper.

## 2. Materials and Methods

**Figure 1:**
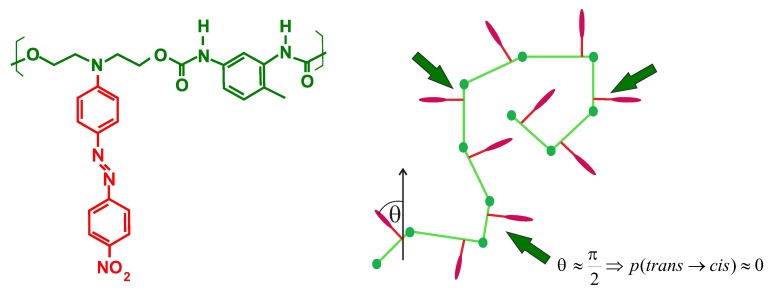
DR1 dye attached to the polymer chain (**left**) and model of chain with dyes, based on bond fluctuation model (**right**). Dyes nearly perpendicular to the light polarization direction do not undergo photoisomerization transition.

**Figure 2:**
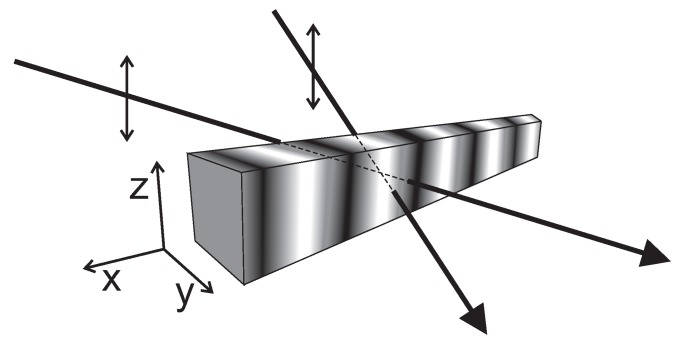
Illumination setup for Degenerated Two Wave Mixing inscription (DTWM) of SRG.

The object of main interest in this study is a polymer chain functionalized with azo-dyes (e.g., DR1, see Figure 1 (left)) in the presence of spatially inhomogeneous light illumination used for an inscription of SRG in DTWM experiment (see Figure 2). The intensity $I_{L}$ of light linearly polarized along *z*-direction, propagating in the *y*-direction, varies along the *x*-direction: $I_{L}=I_{L}\left(x\right)$. The transition rate *p* for trans→cis reaction reads $p\left(x,\theta \right)=\alpha \;I_{L}\left(x\right)\cos^{2}\theta$, where θ stands for an angle between the long axis (transition moment) of trans molecule and the direction of light polarization; the coefficient α accounts for the probability of a photoisomerization reaction in a single act of photon absorption [1]. In what follows, we use the parameter $I\left(x\right)=\alpha \;I_{L}\left(x\right)$, referred to, for simplicity, as (reduced) light intensity.

In the next subsections, we describe the model of a functionalized chain (model material) and the methods used to study its kinetics/dynamics, promoted by multiple trans↔cis photoisomerization cycles of azobenzene dyes attached to the chains.

### 2.1. Polymer Chains Functionalized with Azo-Dyes: Bond Fluctuation Model

Classic Monte Carlo Bond Fluctuation Model in two [42] as well as in three dimensions [39] is one of the most successful statistical physics models of polymer systems. It is a non-specific lattice model for coarse-grained polymer chains, originating from bead-spring model [43,44], which combines methodological simplicity with powerful prediction capabilities. It reproduces a large variety of static and dynamic effects in dense polymer systems, e.g., interdiffusion [39], reptation dynamics [45,46], layers of polymer chains end-grafted placed on surfaces [47,48,49], wetting phenomena [50,51,52], and glass transition in two-dimensional polymer melt [53] (a more detailed review can be found in Refs. [54,55,56]). BF model was also used by our group for modeling nonlinear-optics effects in host (polymer matrix)–guest systems, e.g., dynamics of inscription of diffraction gratings in DNA-based materials [57,58], and in conventional polymer matrices [59,60], inscription of Surface Relief Gratings [38], photomechanical effect [61], second-harmonic generation in poled polymers [62] or all-optical poling of azo-dye guest molecules in polymer matrix [63].

In BFM framework [39], each polymer chain is a system of *N* monomers (representing Kuhn elements) on a simple cubic lattice, connected by bonds. The multitude of bond lengths and angles offers a discrete representation of the continuous-space behavior of real polymer solutions and melts. Six non-equivalent bond orientations with bond lengths equal (in lattice constants) to 2, $\sqrt{5}$, $\sqrt{6}$, 3, 3, and $\sqrt{1}0$ are used. The corresponding bond stretching energies $E_{i}\;\left(i=1,\cdots ,6\right)$ are expressed in the form $E_{i}=E_{0}\;\varepsilon_{i}$, where parameter $E_{0}$ sets an energy scale. In BFM only two values for the energies of the bonds are used: $\varepsilon_{i}=\varepsilon =1$ for the first three bond lengths, and $\varepsilon_{i}$ = 0 for the remaining lengths. The energy $E_{0}$ defines the reduced temperature $T^{*}=k_{B}T/E_{0}$, where $k_{B}$ stands for the Boltzmann constant and *T* for absolute temperature.

Bond fluctuation model was modified by us [38] to account for the functionalization of a polymer chain with azobenzene dyes. The dyes in trans state are assumed to be strictly perpendicular to the bond (Figure 1 (right)).

Two kinds of polymer systems were simulated: (i) single polymer chain system; and (ii) dense polymer matrix. Single polymer chain system, which played the central role in this study, is a statistical ensemble of $N_{0}=10^{3}$ independent polymer chains placed on a $V_{p}=300\times 300\times 300$ simple cubic lattice. In the initial state, center of mass of each chain is placed in the center of the system. While the detailed results are reported for chains with N=25, a summary of the results for various values of *N* is also given. The MC simulations were performed at reduced temperature $T^{*}=0.25$, close to the glass temperature $T_{g}^{*}$ [63]. The polymer matrix system played a minor role in this study and we report here the results calculated on the basis of earlier massive simulations [38] for slightly shorter chains (N=20) at slightly higher temperature $T^{*}=0.3$. The matrix contains *M* = 24,000 polymer chains placed on a $V_{p}=200\times 200\times 200$ simple cubic lattice, which corresponds to a dense polymer melt at reduced density $\rho =8\;M\;N/V_{p}=0.48$ [39].

### 2.2. Monte Carlo Simulations of Functionalized BFM Chains

The polymer chains (single or matrix) are in thermal equilibrium at temperature *T* in the absence of light illumination. The typical configurations can be sampled using Metropolis Monte Carlo (MC) algorithm [64]. To this end, an instantaneous configuration with energy $E_{old}$ undergoes a trial change (trial movement) which yields a trial configuration with energy $E_{new}$. The Metropolis rule accepts the trial configuration as a member of a set of typical equilibrium configurations at temperature *T* with probability equal to the smaller value of two expressions: 1 and $e^{-\left(E_{new}-E_{old}\right)/\left(k_{B}T\right)}$. One MC step (MCS) corresponds to a sweep of trial movements over all the monomers and sets a unit of MC “time” *t* measured in the number of MCS.

In a single MCS each of the monomers performed two kinds of trial movements: driven by thermal fluctuations and non-thermal one, resulting from the interaction with light. The former, performed along one of randomly chosen three directions x,y,z, has unit length. It is accepted if the following three conditions are fulfilled [65]: (i) a length of a trial bond does not violate imposed restrictions; (ii) steric constraints are obeyed; and (iii) the Metropolis acceptance rule does not reject the movement.

Non-thermal trial movements of the monomers reflect the effects of light–matter (polymer chains) interaction. They mimic, in MC simulations, the action of Newtonian forces and torques on the monomers, resulting from trans↔cis photoisomerization cycles of azo-dye molecules. The corresponding generic model, introduced in our paper [38], was used without any modifications in this study, because the objective was to get a deeper insight into the mechanisms that promote light-driven transport of functionalized polymers, reported in the framework of this particular model. It mimics the mechanical impact of trans→cis transition by granting an additional, non-thermal trial movement (of unit length along one of the three directions x,y,z) to the monomer closest to the dye, with probability per unit MCS equal to reduced local light intensity I(x). The trial movement of the monomer is accepted if Conditions (i) and (ii) formulated above are satisfied. The Metropolis acceptance rule (Condition (iii)) is not taken into account because the trial movement is not driven by thermal fluctuations—the typical thermal energy at room temperature $k_{B}T\approx 3\times 10^{-2}$ eV is much smaller than typical energy (a few eV) of light quanta that trigger the photoisomerization transition.

The original model [38], which plays a role of a “minimal” model of light-induced transport of azo-polymers, uses some simplifications in modeling of the photoisomerization cycles. Firstly, it does not account directly for the kinetics of cis→trans transitions: after the photoisomerization transitions the molecules return to trans states. This choice was motivated by the fact that taking into account cis→trans transitions introduces an additional parameter to the model [66], which gives rise to another temporal scale but, on the other hand, does not modify the effect of mass transport in a qualitative way. Secondly, the angular dependence of the transition rate—term $\cos^{2}\left(\theta \right)$—was replaced by a step function with value 0 for a small interval of angles around θ=π/2: π/2-δ<θ<π/2+δ(δ<<1) (see Figure 1 (right)); for the remaining angles, the step function has value 1. This choice was motivated by the fact that $\cos^{2}\theta$ is close to 1 in some interval of angles around θ=0 and is small in some interval around θ=π/2. In the lattice model, the angles θ form a discrete set. The choice of δ from Ref. [38] corresponds, in a continuous model, to a deactivation of photoisomerization transitions for the chromophores with $\delta <18^{\circ }$. To summarize, the transition rate p(x,θ) is either I(x)<1 or 0; the latter applies only when the dye in *trans* state is strictly perpendicular to the light polarization direction.

The selection of the correct length of MC simulation run is crucial since, in general, the type of dynamics of a polymer chain depends on the time interval [43]. The current project was oriented towards the dynamics of polymer chains during the process of inscription of SRG which, according to our previous study [38], requires about $5\times 10^{4}$ MCS. Thus, the same MC interval was used in this study.

### 2.3. Characterization of the Displacement of the Chain

In this paper, we characterize the types of MC dynamics of single chains promoted by various types of light illumination. In what follows, we refer to MC dynamics as dynamics. We analyzed the simplest, but nevertheless informative, characteristic, namely the motion of CM of polymer chains. The procedure is as follows. Center of mass of a chain performs a kind of random walk. The vector ${\vec{r}}_{1}^{\left(CM\right)}\left(t\right)$ of CM of a single chain is calculated after each MC step. This random walk is characterized by the square of the displacement of CM from the initial position at t=0: ${\left(\Delta {\vec{r}}_{1}^{\left(CM\right)}\right)}^{2}\left(t\right)={\left({\vec{r}}_{1}^{\left(CM\right)}\left(t\right)-{\vec{r}}_{1}^{\left(CM\right)}\left(0\right)\right)}^{2}$. Since this parameter strongly fluctuates, we repeated the simulations for a statistical ensemble consisting of $N_{0}=10^{3}$ independent chains and thereafter averaged single squared displacements ${\left(\Delta {\vec{r}}_{i}^{\left(CM\right)}\right)}^{2}\left(t\right),\;i=1,\cdots ,N_{0}$, over this ensemble to obtain the square of the displacement of CM ${\left(\Delta {\vec{r}}^{\left(CM\right)}\right)}^{2}\left(t\right)$, which characterizes the random walk of a single chain:

(1) $${\left(\Delta {\vec{r}}^{\left(CM\right)}\right)}^{2}\left(t\right)=\frac{1}{N_{0}}\sum_{i=1}^{N_{0}}{\left(\Delta {\vec{r}}_{i}^{\left(CM\right)}\right)}^{2}\left(t\right).$$

A similar procedure was applied to the polymer matrix; the averaging was performed over all the chains in the matrix.

In the case when the log-log plot of ${\left(\Delta {\vec{r}}^{\left(CM\right)}\right)}^{2}\left(t\right)$ against *t* is linear, a power law with an exponent γ is present:

(2) $${\left(\Delta {\vec{r}}^{\left(CM\right)}\right)}^{2}\left(t\right)\propto t^{\gamma }.$$

In what follows, we skip, for the sake of simplicity, the symbol (CM), and use the notation ${\left(\Delta r\right)}^{2}$ instead of ${\left(\Delta \vec{r}\right)}^{2}$.

## 3. Results

### 3.1. Anomalous Dynamics: Bulk

**Figure 3:**
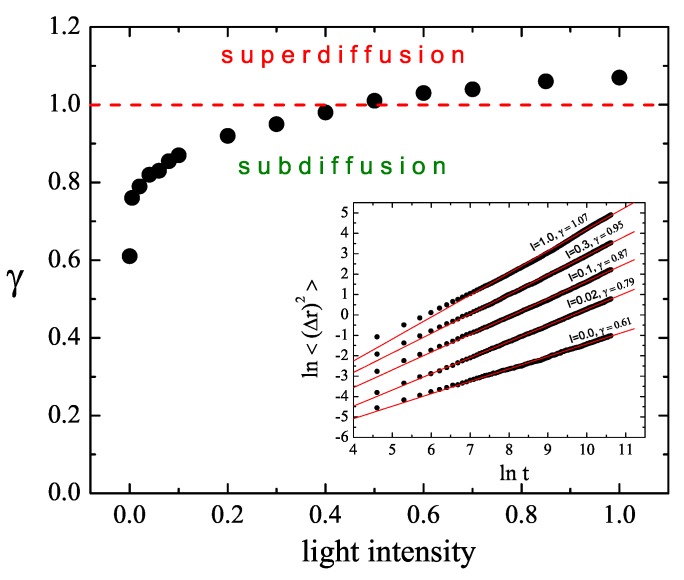
Plot of exponent γ (Equation (2)) as function of the reduced light intensity $I_{0}$, calculated from the plots of $\ln\langle {\left(\Delta r\right)}^{2}\rangle$ against lnt (Inset). Constant illumination. Error bars are of a size of graphical symbols.

**Figure 4:**
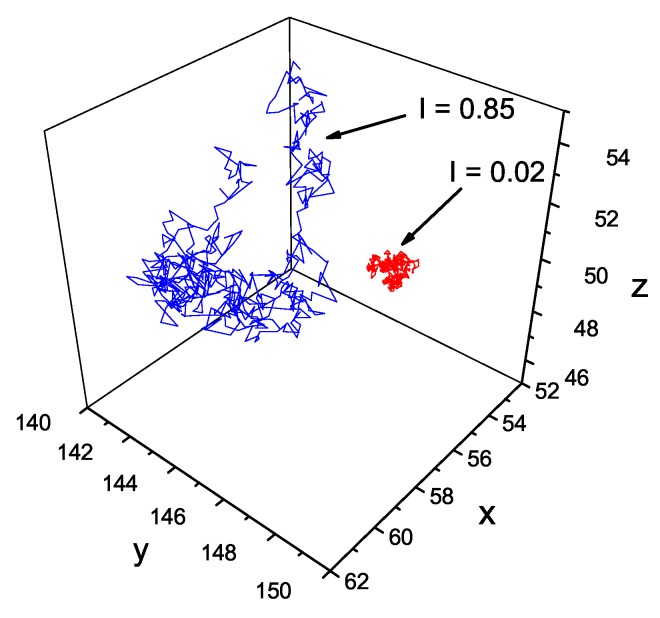
Exemplary trajectories of CM of chains for different illumination intensity, $I_{0}=0.85$ and $I_{0}=0.02$, for a system with constant illumination.

To gain some insight into the type of local light-intensity-dependent dynamics of the chains, we studied the displacement ${\left(\Delta r\right)}^{2}\left(t\right)$ at constant illumination $I=I_{0}$. The results are shown in Figure 3. The log-log plot of ${\left(\Delta r\right)}^{2}\left(t\right)$ against *t* has a linear character, which implies the presence of a power law. For sufficiently low values of $I_{0}$, the system undergoes a sub-diffusion. In particular, a purely polymeric system without illumination shows, as expected [43], a sub-diffusion behavior, with γ≃0.61. With increasing intensity, exponent γ increases and the diffusion becomes normal (γ=1) for intensity $I_{0}$ close to 0.5. For still higher intensities, one finds γ>1, which corresponds to super-diffusion. This light-dependent crossover between all three types of diffusion is briefly discussed in Section 4. The typical trajectories of CM, corresponding to sub-diffusive and super-diffusive motions, are shown in Figure 4—the former is localized in a small volume of the system while the latter displays diffusive as well as ballistic types of motion.

### 3.2. Anomalous Dynamics: Single Chains

#### 3.2.1. Homogeneous Illumination

**Figure 5:**
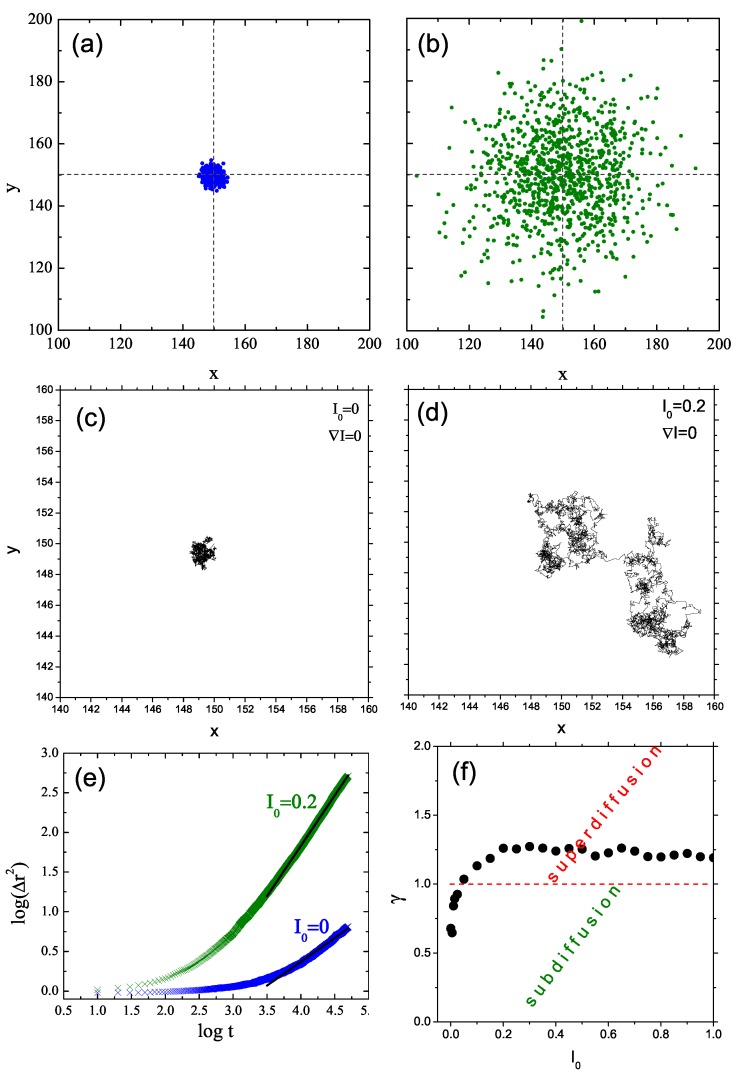
Projection of CM of $10^{3}$ chains onto x-y plane for $I_{0}=0.0$ (**a**) and 0.2 (**b**) at the end of the simulation for homogeneous illumination (∇I=0). Projection of a typical trajectory onto x-y plane for $I_{0}=0.0$ (**c**) and 0.2 (**d**). Log-log plot of ${\left(\Delta r\right)}^{2}\left(t\right)$ for $I_{0}=0.0$ and 0.2 (**e**). Plot of exponent γ as function of intensity $I_{0}$ for homogeneous illumination (**f**).

Complex behavior observed in the bulk at constant illumination has its origin in complex dynamics of single chains at constant illumination. Figure 5a,b shows the projections of CM of $10^{3}$ chains onto x-y plane at the end of the simulation ($5\times 10^{4}$ MCS) for $I_{0}=0.0$ (no illumination) and $I_{0}=0.2$, respectively. In the first case, the CM are localized in a small volume while the end positions of CM for non-zero intensity display a large scatter amounting to at least 25% of linear size of the system. The x-y projections of corresponding exemplary trajectories are shown in Figure 5c,d. In the absence of illumination the trajectory is well-localized. An unexpected result is that at low value of illumination ($I_{0}=0.2$) the trajectory is very different from its counterpart in the bulk (Figure 4) and bears a qualitative similarity to a spatially extended bulk trajectory for $I_{0}=0.85$. In particular, two regions of diffusive-like motion are separated by ballistic-like motion. The log-log plots of displacement ${\left(\Delta r\right)}^{2}\left(t\right)$, shown in Figure 5e, were analyzed using linear fits in the last $4.5\times 10^{4}$ MC steps for various values of $I_{0}$. Plot of exponent γ as function of $I_{0}$ (Figure 5f) displays similar qualitative features as its bulk counterpart (Figure 3), but the threshold intensity which separates the sub-diffusive and super-diffusive regimes is much lower. Actually, the sub-diffusion occurs only at very low values of $I_{0}$.

**Figure 6:**
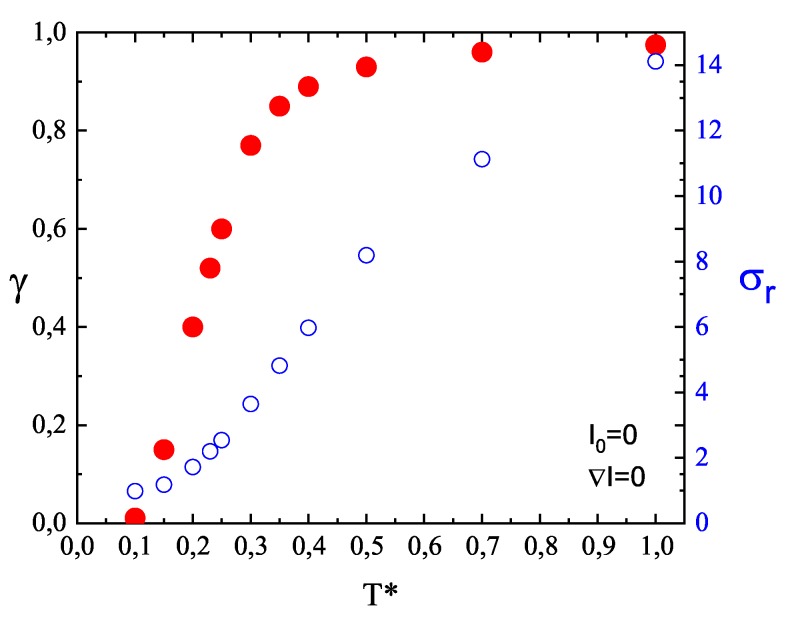
Temperature dependence of exponent γ (red full circles, left axis) and standard deviation $\sigma_{r}$ of displacement of CM of chains (white circles, right axis) for a system of single chains in the absence of illumination.

Exponent γ depends on the temperature. Figure 6 shows the plot of $\gamma (T^{*})$ in the absence of illumination ($I_{0}=0$). In the glassy phase γ<1, and it increases nearly linearly with temperature; above the glass transition temperature another regime sets in, with a weaker increase of this exponent, which becomes close to γ=1. Thus, a crossover from sub-diffusion at low temperature to normal diffusion at high temperature is observed. On the contrary, the standard deviation $\sigma_{r}$ of displacement of CM (Figure 6) does not mark this crossover.

#### 3.2.2. Inhomogeneous Illumination

As expected, the dynamics of chains at constant illumination is isotropic in the space—no chosen direction of motion is present. The driving force for directed motion results from inhomogeneity of the illumination. To ascertain its impact on the dynamics of displacement of CM, we studied linear in *x* light intensity along the *x*-axis:

(3) $$I\left(x\right)=I_{0}-\nabla I\;\left(x-x_{0}\right),$$

where $x_{0}$ denotes center of lattice in the *x* direction ($x_{0}=150$) and $I_{0}=I\left(x_{0}\right)$ is the intensity offset. The coefficient of proportionality $\nabla I\equiv \frac{\partial I}{\partial x}$ is referred to as gradient, because in the illumination setup used in this study the only non-zero component of gradient of light intensity is its *x*-component.

**Figure 7:**
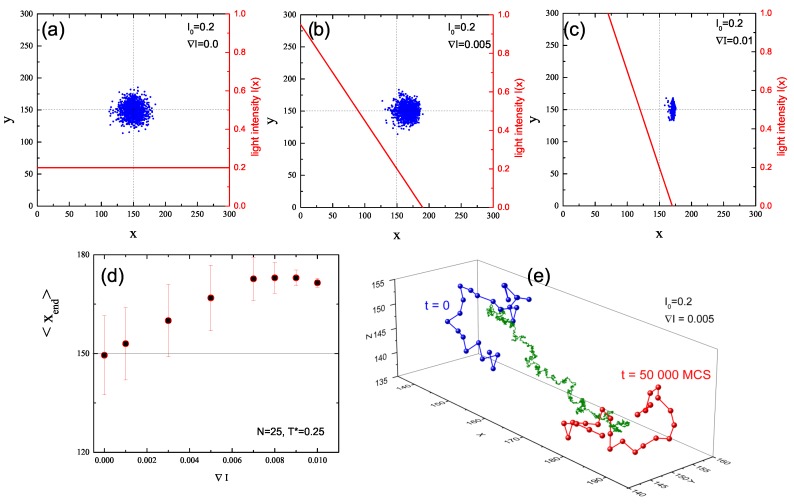
Projection of CM of $10^{3}$ chains onto x-y plane at the end of the simulation for $I_{0}=0.2$ and ∇I=0 (**a**), ∇I=0.005 (**b**), and ∇I=0.01 (**c**). Red lines are plots of I(x), Equation (3) (right axis). Plot of averaged position $<x_{end}>$ of CM and its standard deviation (**d**). Exemplary initial (t=0, blue circles) and final ($t=5\times 10^{4}$ MCS, red circles) configurations of the chain and trajectory of CM during illumination phase (green points) for $I_{0}=0.2$ and ∇I=0.005 (**e**).

The effect of light-gradient-driven directed movement of polymer chains from bright to dark places is shown in Figure 7, which displays the x-y projections of CM of polymer chains at the end of the simulation. Intensity offset $I_{0}$ is fixed. When the gradient vanishes (Case (a)) the distribution of CM is isotropic around the center of coordinate frame. When the gradient becomes non-zero a systematic shift to the right appears (Case (b)). For still larger values of the gradient, the distribution becomes flat around x=170 (Case (c)). This effect reflects the fact that I(x) vanishes at this point, and the directed movements of all the chains which have reached this point suddenly stop. The dependence of this shift, defined as average (over chains) $<x_{end}>$ position of CM at the end of simulation, on intensity gradient is presented in Figure 7d. Two regimes are present. The first one corresponds to a linear increase of the shift:

(4) $$<x_{end}>\;\propto \nabla I.$$

Since $<x_{end}>$ divided by the time of the motion is the average velocity $<v_{CM}>$ of the CM of the chains, we found that

(5) $$<v_{CM}>\;\propto \nabla I.$$

This result is briefly discussed in Section 4. The second regime, where $<x_{end}>$ is approximately constant, results from the above discussed vanishing of the gradient and does not correspond to any new dynamical effect. An exemplary trajectory of CM during the illumination period (as well as initial and final configurations of the chain) in the first regime is shown in Figure 7e. It displays, on a large scale, a linear character superimposed, on much shorter scales, with some kind of random walk.

**Figure 8:**
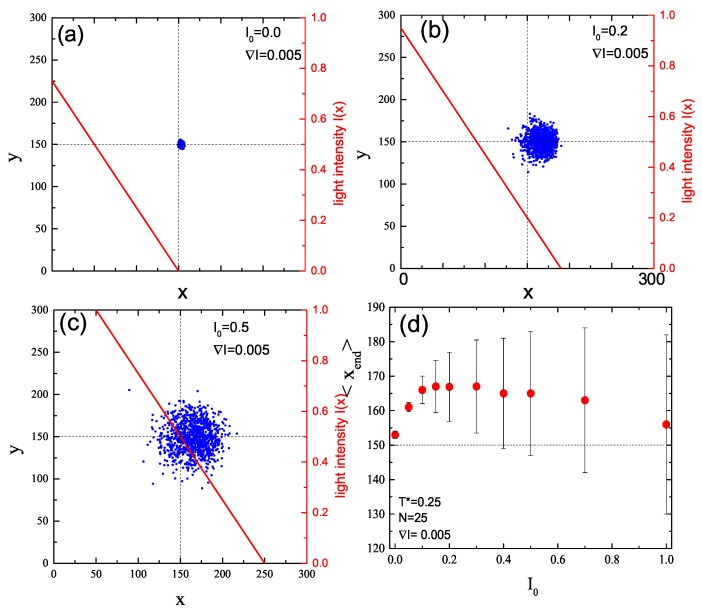
Projections of CM of $10^{3}$ chains onto x-y plane at the end of the simulation for constant value ∇I=0.005 and $I_{0}=0$ (**a**), $I_{0}=0.2$ (**b**), and $I_{0}=0.5$ (**c**). Red lines are plots of I(x), Equation (3) (right axis). Averaged displacement of CM of chains in *x* direction and its standard deviation as a function of $I_{0}$ (**d**).

Impact of intensity offset $I_{0}$ on directed displacement driven by constant intensity gradient (∇I=0.005) is characterized in Figure 8. When the offset is missing (Figure 8a), the directed motion is weak and the spread of CM at the end of the illumination period is small. Non-zero intensity offset $I_{0}=0.2$ enhances both parallel (along gradient of intensity) and perpendicular motions (Figure 8b). For still larger values of the offset, $I_{0}=0.05$ (Figure 8c), the isotropic motion starts to suppress the average directed motion. This enhancement/attenuation effect is summarized in Figure 8d, which shows that: (i) the plot of $<x_{end}>$ in function of $I_{0}$ has a maximum; and (ii) its standard deviation increases, suppressing the average directed motion.

Since the gradient of light intensity is non-zero only in *x* direction, it is reasonable to study the scaling of the mean square displacement of CM with time separately for *x* (parallel) and y,z (transverse) directions. Instead of Equation (2), we have

(6) $${\left(\Delta x\right)}^{2}\left(t\right)\propto t^{\gamma_{x}},\;{\left(\Delta y\right)}^{2}\left(t\right)\propto t^{\gamma_{y}},\;{\left(\Delta z\right)}^{2}\left(t\right)\propto t^{\gamma_{z}}.$$

**Figure 9:**
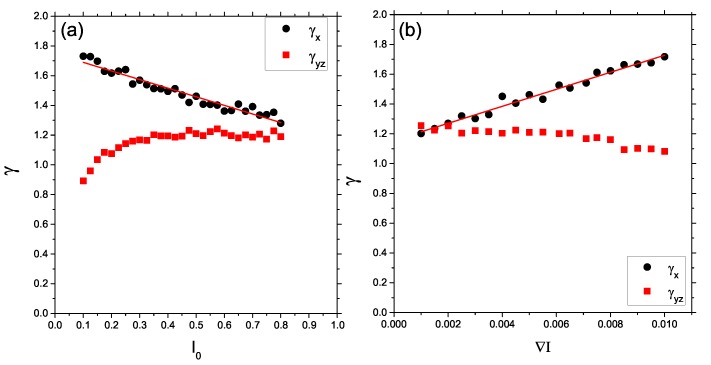
Plot of exponents $\gamma_{x}$ (black circles) and $\gamma_{y,z}$ (red squares) as functions of intensity offset $I_{0}$ for ∇I=0.005 (**a**) and of gradient of intensity ∇I for $I_{0}=0.5$ (**b**).

Figure 9 (left) shows the plots of exponents $\gamma_{x}$ (black circles) and $\gamma_{y,z}$ (red squares) in function of intensity offset $I_{0}$. As expected, $\gamma_{x}\neq \gamma_{y}\approx \gamma_{z}$. Parallel motion of CM is super-diffusive; $\gamma_{x}$ decreases linearly as offset intensity increases. We ascribe this effect to the competition between purely directed motion driven by the gradient of the intensity and isotropic motion in the absence of gradient of illumination. When the value of offset $I_{0}$ increases, the contribution of directed motion to an overall motion decreases—for large values of $I_{0}$, we found $\gamma_{x}\approx 1.25$, which is close to γ for constant illumination (see Figure 5f). This interpretation is supported by the fact that, for large values of $I_{0}$, the three exponents have similar values: $\gamma_{x}\approx \gamma_{y}\approx \gamma_{z}$. Rather surprisingly, the transverse motion also displays super-diffusive motion. Figure 9 (right) shows the dependence of the exponents on the gradient of intensity ∇I for $I_{0}=0.5$. For very low gradients, the exponents are again approximately equal. The exponents for transverse motion are weakly dependent on ∇I, while $\gamma_{x}$ increases linearly with ∇I. This increase reflects the enhancement of the role of directed motion in comparison with an isotropic motion.

#### 3.2.3. Role of Chain’s Length

**Figure 10:**
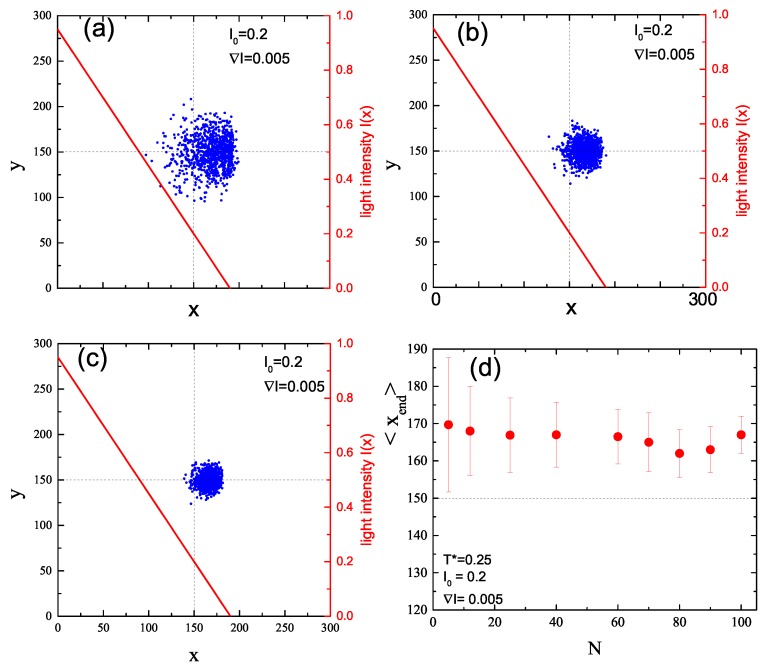
Projection of CM of $10^{3}$ chains onto x-y plane at the end of the simulation for $I_{0}=0.2$, ∇I=0.005 and different number of monomers: N=5 (**a**); N=25 (**b**); and N=60 (**c**). Averaged displacement $<x_{end}>$ of CM of chains in *x* direction and its standard deviation as a function of *N* (**d**).

One expects that the length of the chain can modify the type of its dynamics. Figure 10 characterizes the end positions of CM of chains with lengths N=5, 25 and 60 (Figure 10a–c, respectively) for $I_{0}=0.2$ and ∇I=0.005. Those results are summarized in Figure 10d, which shows the plot of $<x_{end}>$ in function of *N*. A rather unexpected result is that this displacement is weakly dependent on chain’s length. On the contrary, its standard deviation systematically decreases as *N* increases, which corresponds to a more compact localization of positions of CM presented in Figure 10a–c.

**Figure 11:**
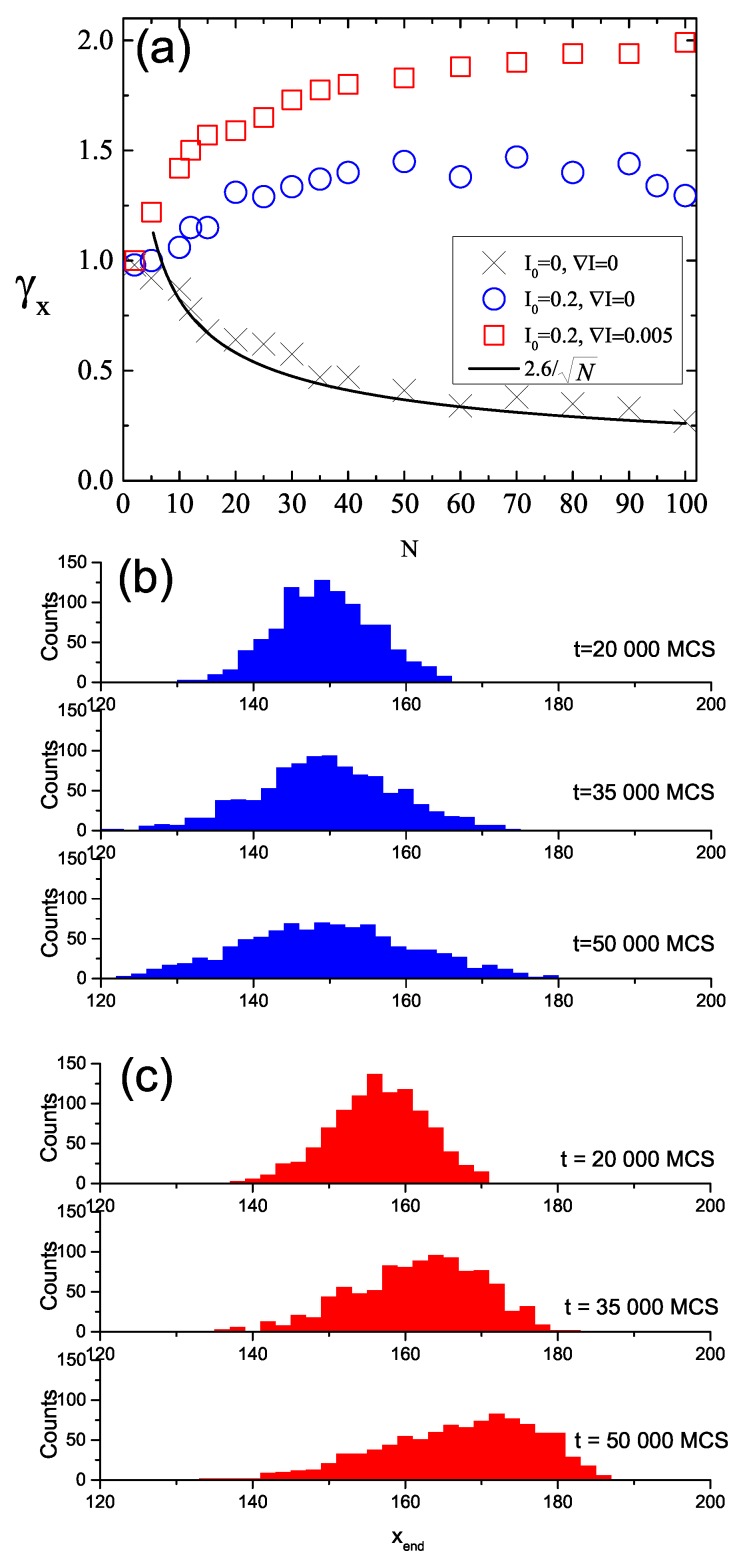
Exponent $\gamma_{x}$ as function of chain’s length *N* for the system without illumination (black crosses), homogeneous illumination with $I_{0}=0.2$ (blue circles) and inhomogeneous illumination with ∇I=0.005 (red squares). The black solid line fits the data using function $N^{-1/2}$ (**a**). Histograms of instantaneous positions of CM of chains during the illumination phase for homogeneous illumination with $I_{0}=0.2$ (**b**) and inhomogeneous illumination with ∇I=0.005 (**c**).

Scaling of ${\left(\Delta r\right)}^{2}$ with time, characterized by exponent $\gamma_{x}$, Equation (6), leads to interesting observations. Figure 11a shows the plots of $\gamma_{x}\left(N\right)$ for three characteristic cases: systems without illumination ($I_{0}=0,\nabla I=0$, black crosses), with constant illumination ($I_{0}=0.2,\nabla I=0$, blue circles) and with both offset illumination and gradient ($I_{0}=0.2,\nabla I=0.005$, red squares)—the latter corresponds to data shown in Figure 10.

In the absence of illumination, a sub-diffusive regime is present: $\gamma_{x}<1$ and it decreases as *N* increases. This kind of behavior is well-known in polymer physics [43]. The black solid line, which is the plot of the function

(7) $$\gamma_{x}\propto N^{-1/2},$$

reasonably reproduces simulation data. We point out that Equation (7) characterizes the motion of CM of polymer chains in Zimm model [43], which takes into account hydrodynamic interactions.

Constant illumination promotes, in general, a super-diffusive motion, but the dynamics of short chains (e.g., with N<10) is close to that of a standard diffusion. For longer chains (e.g., N>40) exponent $\gamma_{x}$ becomes weakly dependent on chain’s length. Finally, in the case when both offset and gradient of illumination are present a new effect appears. Namely, the value of $\gamma_{x}$ for the longest chains becomes close to 2, which corresponds to the ballistic diffusion. The origin of this effect is as follows. Figure 11b,c show the histograms of instantaneous positions of CM of chains during simulations for homogeneous illumination with $I_{0}=0.2$ and inhomogeneous illumination with ∇I=0.005, respectively. In the former case, a symmetric dispersion of CM positions is present, while, for a system with intensity gradient, a systematic drift is present in addition to the dispersion. The superposition of the drift and dispersion promotes a nearly ballistic motion.

### 3.3. Continuous Time Random Walk Model for Inscription of Surface Relief Grating

The results presented above give a firm ground to establish a physical picture of the dynamics of polymer chains in the process of SRG inscription as corresponding to a dynamic coexistence of local sub-diffusive, diffusive and super-diffusive regimes. This, in turn, offers a possibility of a simple stochastic modeling photoinduced mass transport in polymer physics and, in particular, nonlinear optics phenomena in host–guest systems. In what follows, we propose such a “toy” model of inscription of SRG.

Anomalous dynamics can be studied using either fractional diffusion equations or Continuous Time Random Walk (CTRW) [67]. We use the latter, which is a non-trivial generalization of a standard random walk with Levy α-stable distribution of jumps in space and a one-parameter (β) Mittag–Leffler distribution of waiting times between the jumps. The squared displacement has scaling form [67]:

(8) $${\left(\Delta r\right)}^{2}\left(t\right)\propto t^{-\delta },\;\delta =\frac{2\beta }{\alpha }.$$

**Figure 12:**
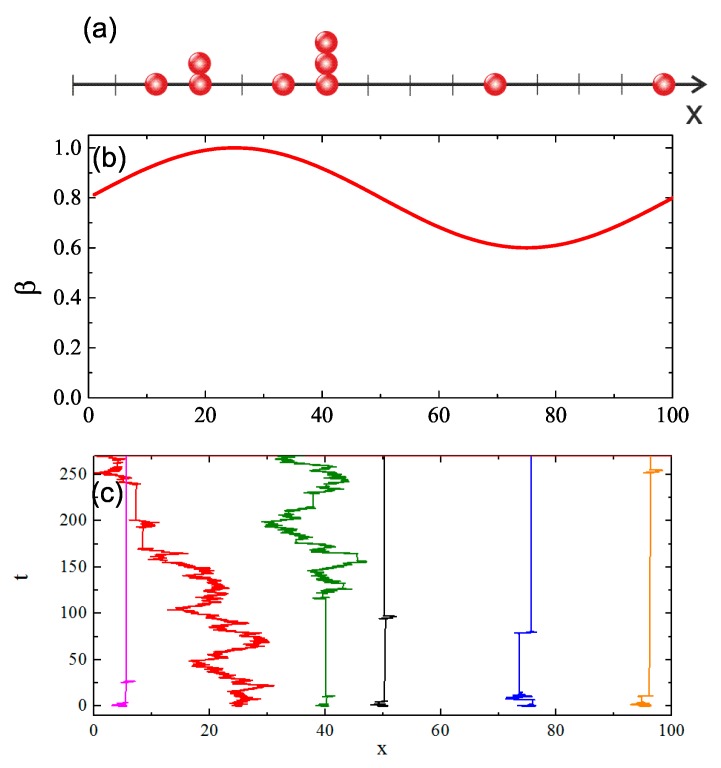
Independent CTRW walkers on a line (**a**). Spatial modulation of parameter β=β(x) of Mittag-Leffler distribution of waiting times (**b**). Exemplary trajectories for a few walkers performing CTRW with α=2 and β=β(x) (**c**).

The model reduces the motion of chains in 3D to the motion of a system of independent walkers on a line (see Figure 12a). Each walker represents the CM of a chain and performs CTRW with α=2—the jumps along *x*-axis are then distributed according to the gaussian probability density. In this case, the type of an anomalous dynamics depends on value of parameter β: it is sub-diffusive for β<1, diffusive for β=1 and super-diffusive for β>1. This classification coincides with its counterpart based on parameter γ. Thus, we put

(9) β=γ.

Since γ=γ(x), our model corresponds to a modification of CTRW for a system of independent walkers with parameter β which depends on an actual position of the walker. We studied a system of $10^{6}$ walkers on an interval of *x*-axis, which corresponds to a full period of sinusoidally modulated intensity I(x) (Figure 12a), typical for DTWM inscription of SRG. For simplicity, we modulated parameter β(x) accordingly (see Figure 12b). We chose β(x)≤1, motivated by values of γ found for the bulk system (Figure 3). Random walks were generated using Monte Carlo approach proposed in Ref. [68]. Exemplary trajectories for a few values of parameter β(x) are shown in Figure 12c. The trajectory which starts from x=25 (which corresponds to the maximum of β and also to the maximum of the illumination) moves away towards less illuminated regions, in agreement with experimental observations. The trajectories corresponding to sub-diffusive regime display a large amount of dynamical arrest.

**Figure 13:**
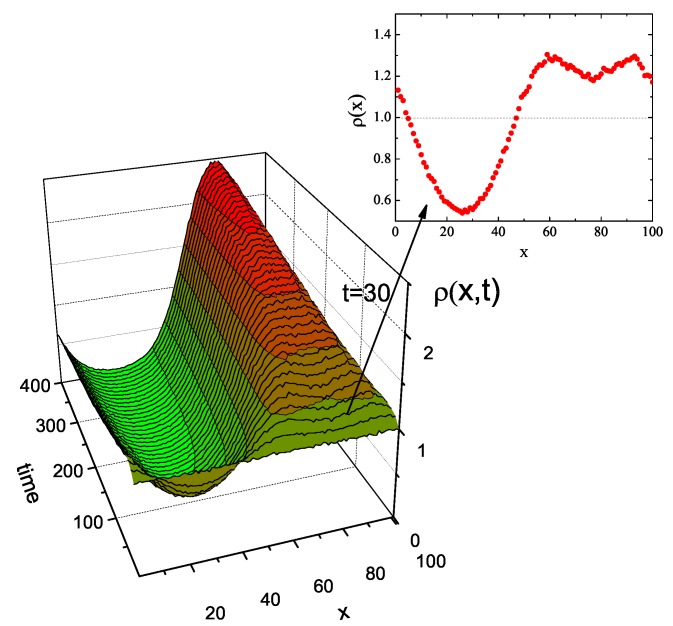
Temporal evolution of density ρ(x,t) of system of walkers performing CTRW with α=2 and β=β(x). See text for more details.

The results of MC simulations of the density ρ(x,t) of the walkers in the neighborhood of point *x* at time *t* are presented in Figure 13. We found, in agreement with the experimental observations, that the walkers (“polymers”) move away from bright places and move towards dark places. Surprisingly, the model reproduces the fine structure (two sub-peaks) of the main density peak, as found in experiments [40,41] and in MC modeling [38]. The fine structure is a transient effect, corresponding to one of the scenarios discussed in Ref. [38]. A more detailed report on stochastic modeling will be presented elsewhere.

## 4. Discussion

A partial, rather technical discussion of the results accompanies the presentation of the results. Here, we discuss them in a wider context.

We characterized the types of Monte Carlo dynamics of photoinduced motion of model polymer chains functionalized with azo-dyes using a kinetic Monte Carlo model introduced in our earlier paper [38]. We implemented the lattice bond fluctuation model, used extensively in the simulations of polymer systems. The length of MC runs corresponded to the typical period of MC inscription of model SRG [38]. It is imperative to point out that the results of current study cannot be extrapolated onto arbitrary long MC “time” intervals.

The most important, rather unexpected, result is that the chains—single as well as their assembly—can display sub-diffusive, diffusive and super-diffusive regimes of motion, depending on the parameters of illumination. The types of diffusion—normal or anomalous—are characterized by an exponent γ in the power-law dependence of the average squared displacement of the center of mass of a chain on MC “time”, see Equation (2). For constant illumination, a crossover from sub-diffusion (γ<1) to super-diffusion (γ>1) takes place at some threshold value of light intensity; no directed motion was found. Super-diffusion is stronger for a single chain than for an assembly of chains. On the contrary, the directed motion of the chains results from an inhomogeneous illumination and it is always super-diffusive in the direction of the gradient. The motion in perpendicular direction can be sub-diffusive, diffusive or super-diffusive.

We quantified, to some extent, the relations between the displacement of the chain and the light illumination. Namely, the distance covered by the center of mass of a chain in the simulation is, on average, proportional to the gradient of illumination. The relation $v\propto -\frac{dI}{dt}$, found previously for a system of polymers [38], holds also for single chains. When both constant offset of the illumination and the gradient of intensity are present, the effect of directed motion becomes weaker. Those results open the possibility of a quantitative interpretation of experiments in the optical control of single functionalized nano-particles through light intensity gradients [69]. This study is in progress now—first results in two dimensions are reported in Ref. [70].

The analysis of the displacement of chains with various lengths yields some rather counter-intuitive results. In the presence of inhomogeneous illumination, the super-diffusive dynamics of the center of mass of the chains becomes more pronounced (has larger values of exponent $\gamma_{x}$, Equation (6)) for longer chains. In particular, the super-diffusive motion along the intensity gradient becomes nearly ballistic for sufficiently long chains. On the other hand, in the absence of light illumination, and in the period of “time” characteristic for the inscription of SRG, $\gamma_{x}$ displays the dependence on *N* as in hydrodynamic Zimm model and not as in Rouse model [43].

The qualitative and quantitative results presented in this paper produce a simple but, at the same time, attractive physical picture of the dynamics of functionalized polymer chains in the process of inscription of Surface Relief Gratings. Namely, polymer chains in various parts of the system undergo normal or anomalous (sub- or super-diffusion) dynamics, depending on the local value of light illumination. Those parts are in a dynamical coexistence. This is, to the best of our knowledge, the first sound physical model which exhibits, at the same time, spatially separated normal and anomalous diffusion.

Anomalous dynamics of CM of the chains can be easily modeled using a generalization of a standard random walk, namely continuous time random walk. We proposed and studied a “toy” model for inscription of SRG, where the role of the functionalized polymer chains was played by a system of non-interacting CTRW walkers. This simple model reproduces not only the transport of mass from bright to dark places but also, rather unexpectedly, a controversial experimental effect: transient fine structure of SRG, reported also in massive simulations of our MC model [38]. Systematic studies of CTRW model are in progress now.

The anomalous dynamics of single chains was characterized for various illumination setups. The next step in a study of a photoinduced dynamics is a detailed statistical analysis of light-induced transitions of azo-dyes attached to the chain and of the impact of their dynamics on the anomalous diffusion. This topic, which goes beyond the scope of this paper, is in progress now.

## 5. Conclusions

There are two main conclusions of this study. Firstly, the model of light-induced motion of polymer chains functionalized with azo-dyes introduced in our earlier paper [38] in the context of inscription of Surface Relief Grating becomes a promising candidate for a more general study of complex dynamics of light-induced motion of a large variety of azo-materials. Secondly, it becomes evident that models of light-driven transport of azo-materials based on normal diffusion are inadequate. Instead, more advanced methods of modeling of anomalous diffusion, e.g., continuous time random walk, stochastic differential equations, or fractional diffusion equations, should be used.

## References

[1] Sekkat Z., Knoll W. (2002). Photoreactive Organic Thin Films.

[2] Kim D.Y., Tripathy S.K., Li L., Kumar J. (1995). Laser-induced holographic surface relief gratings on nonlinear optical polymer films. Appl. Phys. Lett..

[3] Rochon P., Batalla E., Natansohn A. (1995). Optically induced surface gratings on azoaromatic polymer films. Appl. Phys. Lett..

[4] Lee S., Kang H.S., Park J.-K. (2012). Directional photofluidization lithography: Micro/ nanostructural evolution by photofluidic motions of azobenzene materials. Adv. Mater..

[5] Mahimwalla Z., Yager K.G., Mamiya J., Shishido A., Priimagi A., Barrett C.J. (2012). Azobenzene photomechanics: Prospects and potential applications. Polym. Bull..

[6] Pedersen T.G., Johansen P.M., Holme N.C.R., Ramanujam P.S., Hvilosted S. (1998). Mean-field theory of photoinduced formation of surface reliefs in side-chain azobenzene polymers. Phys. Rev. Lett..

[7] Barrett C.J., Natansohn A.L., Rochon P.L. (1996). Mechanism of optically inscribed high-efficiency diffraction gratings in azo polymer films. J. Phys. Chem..

[8] Barrett C.J., Rochon P.L., Natansohn A.L. (1998). Model of laser-driven mass transport in thin films of dye-functionalized polymers. J. Chem. Phys..

[9] Yager K.G., Barrett C.J. (2006). Photomechanical surface patterning in azo-polymer materials. Macromolecules.

[10] Henneberg O., Geue T., Saphiannikova M., Pietsch U., Rochon P., Natansohn A. (2001). Formation and dynamics of polymer surface relief gratings. Appl. Surf. Sci..

[11] Lefin P., Fiorini C., Nunzi J.M. (1998). Anisotropy of the photoinduced translation diffusion of azo-dyes. Opt. Mater..

[12] Kumar J., Li L., Jiang X.L., Kim D.-Y., Lee T.S., Tripathy S. (1998). Gradient force: The mechanism for surface relief grating formation in azobenzene functionalized polymers. Appl. Phys. Lett..

[13] Ashkin A., Dziedzic J., Bjorkholm J.E., Chu J.E. (1986). Observation of a single-beam gradient force optical trap for dielectric particles. Opt. Lett..

[14] Sumaru K., Yamanaka T., Fukuda T., Matsuda H. (1999). Photoinduced surface relief gratings on azopolymer films: Analysis by a fluid mechanics model. Appl. Phys. Lett..

[15] Sumaru K., Fukuda T., Kimura T., Matsuda H., Yamanaka T. (2002). Photoinduced surface relief formation on azopolymer films: A driving force and formed relief profile. J. Appl. Phys..

[16] Bellini B., Ackermann J., Klein H., Grave C., Dumas P., Safarov V. (2006). Light-induced molecular motion of azobenzene-containing molecules: A random-walk model. J. Phys. Condens. Matter.

[17] Juan M.L., Plain J., Bachelot R., Royer P., Gray S.K., Wiederrecht G.P. (2009). Multiscale model for photoinduced molecular motion in azo polymers. ACS Nano.

[18] Juan M.L., Plain J., Bachelot R., Royer P., Gray S.K., Wiederrecht G.P. (2008). Stochastic model for photoinduced surface relief grating formation through molecular transport in polymer films. Appl. Phys. Lett..

[19] Plain J., Wiederrecht G.P., Gray S.K., Royer P., Bachelot R. (2013). Multiscale optical imaging of complex fields based on the use of azobenzene nanomotors. J. Phys. Chem. Lett..

[20] Karageorgiev P., Neher D., Schulz B., Stiller B., Pietsch U., Giersig M., Brehmer L. (2005). From anisotropic photo-fluidity towards nanomanipulation in the optical near-field. Nat. Mater..

[21] Mechau N., Saphiannikova M., Neher D. (2005). Dielectric and mechanical properties of azobenzene polymer layers under visible and ultraviolet irradiation. Macromolecules.

[22] Mechau N., Saphiannikova M., Neher D. (2006). Molecular tracer diffusion in thin azobenzene polymer layers. Appl. Phys. Lett..

[23] Srikhirin T., Laschitsch A., Neher D., Johannsmann D. (2000). Light-induced softening of azobenzene dye-doped polymer films probed with quartz crystal resonators. Appl. Phys. Lett..

[24] Fang G.J., Maclennan J.E., Yi Y., Glaser M.A., Farrow M., Korblova E., Walba D.M., Furtak T.E., Clark N.A. (2013). Athermal photofluidization of glasses. Nat. Commun..

[25] Hurduc N., Donose B.C., Macovei A., Paius C., Ibanescu C., Scutaru D., Hamel M., Branza-Nichita N., Rocha L. (2014). Direct observation of athermal photofluidisation in azopolymer films. Soft Matter.

[26] Saphiannikova M., Toshchevikov V. (2015). Optical deformations of azobenzene polymers: Orientation approach vs. photofluidization concept. J. Soc. Inf. Disp..

[27] Toshchevikov V., Saphiannikova M., Heinrich G. (2009). Microscopic Theory of Light-Induced Deformation in Amorphous Side-Chain Azobenzene Polymers. J. Phys. Chem. B.

[28] Toshchevikov V., Saphiannikova M., Heinrich G. (2012). Light-Induced Deformation of Azobenzene Elastomers: A Regular Cubic Network Model. J. Phys. Chem. B.

[29] Toshchevikov V., Saphiannikova M., Heinrich G. (2012). Theory of Light-Induced Deformation of Azobenzene Elastomers: Influence of Network Structure. J. Chem. Phys..

[30] Toshchevikov V., Saphiannikova M. (2014). Theory of Light-Induced Deformation of Azobenzene Elastomers: Effects of the Liquid- Crystalline Interactions and Biaxiality. J. Phys. Chem. B.

[31] Petrova T., Toshchevikov V., Saphiannikova M. (2015). Light-Induced Deformation of Polymer Networks Containing Azobenzene Chromophores and Liquid Crystalline Mesogens. Soft Matter.

[32] Ilnytskyi J.M., Neher D., Saphiannikova M. (2011). Opposite Photo- Induced Deformations in Azobenzene-Containing Polymers with Different Molecular Architecture: Molecular Dynamics Study. J. Chem. Phys..

[33] Ilnytskyi J.M., Saphiannikova M., Neher D., Allen M.P. (2016). Computer Simulation of Side-Chain Liquid Crystal Polymer Melts and Elastomers. Liquid Crystalline Polymers.

[34] Toshchevikov V., Petrova T., Saphiannikova M. (2017). Kinetics of light-induced ordering and deformation in LC azobenzene-containing materials. Soft Matter.

[35] Yadavalli N.S., Linde F., Kopyshev A., Santer S. (2013). Soft matter beats hard matter: Rupturing of thin metallic films induced by mass transport in photosensitive polymer films. ACS Appl. Mater. Interfaces.

[36] Di Florio G., Brundermann E., Yadavalli N.S., Santer S., Havenith M. (2014). Graphene multilayer as nanosized optical strain gauge for polymer surface relief gratings. Nano Lett..

[37] Toshchevikov V., Ilnytskyi J., Saphiannikova M. (2017). Photoisomerization kinetics and mechanical stress in azobenzene-containing materials. J. Phys. Chem. Lett..

[38] Pawlik G., Miniewicz A., Sobolewska A., Mitus A.C. (2014). Generic stochastic Monte Carlo model of the photoinduced mass transport in azo-polymers and fine structure of Surface Relief Gratings. Europhys. Lett..

[39] Deutsch H.P., Binder K. (1991). Interdiffusion and self-diffusion in polymer mixtures: A Monte Carlo study. J. Chem. Phys..

[40] Schab-Balcerzak E., Siwy M., Kawalec M., Sobolewska A., Chamera A., Miniewicz A. (2009). Synthesis, characterization, and study of photoinduced optical anisotropy in polyimides containing side azobenzene units. J. Phys. Chem. A.

[41] Fabbri F., Garrot D., Lahlil K., Boilot J.P., Lassailly Y., Peretti J. (2011). Evidence of two distinct mechanisms driving photoinduced matter motion in thin films containing azobenzene derivatives. J. Phys. Chem. B.

[42] Carmesin I., Kremer K. (1988). The bond fluctuation method: A new effective algorithm for the dynamics of polymers in all spatial dimensions. Macromolecules.

[43] Doi M., Edwards S.F. (1986). The Theory of Polymer Dynamics.

[44] Kawakatsu T. (2004). Statistical Physics of Polymers. An Introduction.

[45] Paul W., Binder K., Heermann D.W., Kremer K. (1991). Dynamics of polymer solutions and melts. Reptation predictions and scaling of relaxation times. J. Chem. Phys..

[46] Jentzsch C., Dockhorn R., Sommer J.-U. (2016). A Highly Parallelizable Bond Fluctuation Model on the Body-Centered Cubic Lattice. Parallel Processing and Applied Mathematics.

[47] Lai P.Y., Binder K. (1991). Structure and dynamics of grafted polymer layers: A Monte Carlo simulation. J. Chem. Phys..

[48] Lai P.Y., Halperin A. (1991). Polymer brush at high coverage. Macromolecules.

[49] Lai P.Y., Zhulin E.Z. (1992). Monte Carlo test of the self-consistent field theory of a polymer brush. J. Phys. II.

[50] Wang J.S., Binder K. (1991). Wetting transitions in polymer blends: A Monte Carlo lattice simulation. J. Chem. Phys..

[51] Wang J.S., Binder K. (1992). Chain linear dimensions in the surface-enriched layer of polymer mixtures. Makromol. Chem. Theory Simul..

[52] Wang J.S., Binder K. (1991). Enrichment of the chain ends in polymer melts at interfaces. J. Phys. I.

[53] Wittmann H.-P., Kremer K., Binder K. (1992). Glass transition of polymer melts: A twodimensional Monte Carlo study in the framework of the bond uctuation method. J. Chem. Phys..

[54] Wittmer J.P., Cavallo A., Kreer T., Baschnagel J., Johner A. (2009). A finite excluded volume bond-fluctuation model: Static properties of dense polymer melts revisited. J. Chem. Phys..

[55] Baschnagel J., Wittmer J.P., Meyer H., Attig N. (2004). Computational Soft Matter: From Synthetic Polymers to Proteins.

[56] Mueller M., Yip S. (2005). Handbook of Materials Modeling.

[57] Pawlik G., Mitus A.C., Mysliwiec J., Miniewicz A., Grote J.G. (2010). Photochromic dye semi-intercalation into DNA-based polymeric matrix: Computer modeling and experiment. Chem. Phys. Lett..

[58] Pawlik G., Radosz W., Mitus A.C., Mysliwiec J., Miniewicz A., Kajzar F., Rau I. (2014). Holographic grating inscription in DR1: DNA-CTMA thin films: The puzzle of time scales. Cent. Eur. J. Chem..

[59] Pawlik G., Mitus A.C., Miniewicz A., Kajzar F. (2004). Monte Carlo simulations of temperature dependence of the kinetics of diffraction gratings formation in a polymer matrix containing azobenzene chromophores. J. Nonlinear Opt. Phys. Mater..

[60] Pawlik G., Mitus A.C., Miniewicz A., Sobolewska A., Kajzar F. (2005). Temperature dependence of the kinetics of diffraction gratings formation in a polymer matrix containing azobenzene chromophores: Monte Carlo simulations and experiment. Mol. Cryst. Liq. Cryst..

[61] Pawlik G., Orlik R., Radosz W., Mitus A.C., Kuzyk M.G. (2012). Towards understanding the photomechanical effect in polymeric fibers: Analysis of free volume in a model polymeric matrix. Proc. SPIE.

[62] Pawlik G., Rau I., Kajzar F., Mitus A.C. (2010). Second-harmonic generation in poled polymers: Pre-poling history paradigm. Opt. Express.

[63] Radosz W., Pawlik G., Mitus A.C. (2018). Complex Dynamics of Photo-Switchable Guest Molecules in All-Optical Poling Close to the Glass Transition: Kinetic Monte Carlo Modeling. J. Phys. Chem. B.

[64] Landau D.P., Binder K. (2000). A Guide to Monte Carlo Simulations in Statistical Physics.

[65] Binder K. (1995). Monte Carlo and Molecular Dynamics Simulations in Polymer Science.

[66] Pawlik G., Mitus A.C., Miniewicz A., Kajzar F. (2003). Kinetics of diffraction gratings formation in a polymer matrix containing azobenzene chromophores: Experiments and Monte Carlo simulations. J. Chem. Phys..

[67] Metzler R., Klafter J. (2000). The random walk’s guide to anomalous diffusion: A fractional dynamics approach. Phys. Rep..

[68] Fulger D., Scalas E., Germano G. (2008). Monte Carlo simulation of uncoupled continuous-time random walks yielding a stochastic solution of the space-time fractional diffusion equation. Phys. Rev. E.

[69] Abid J.-P., Frigoli M., Pansu R., Szeftel J., Zyss J., Larpent C., Brasselet S. (2011). Light-driven directed motion of azobenzene-coated polymer nanoparticles in an aqueous medium. Langmuir.

[70] Wysoczanski T., Mitus A.C., Radosz W., Pawlik G. (2017). Photoinduced (anomalous) dynamics of functionalized polymer chains: Applications for Surface Relief Grating modelling. Proc. SPIE.

